# Short- and Long-Term Major Cardiovascular Adverse Events in Carotid Artery Interventions: A Nationwide Population-Based Cohort Study in Taiwan

**DOI:** 10.1371/journal.pone.0121016

**Published:** 2015-03-24

**Authors:** Ming-Lung Tsai, Chun-Tai Mao, Dong-Yi Chen, I-Chang Hsieh, Ming-Shien Wen, Tien-Hsing Chen

**Affiliations:** 1 Division of Cardiology, Department of Internal Medicine, Chang Gung Memorial Hospital, Taipei, Taiwan; 2 Heart Failure Center, Division of Cardiology, Department of Internal Medicine, Chang Gung Memorial Hospital, Keelung, Taiwan; 3 College of Medicine, Chang Gung University, Taoyuan, Taiwan; Osaka University Graduate School of Medicine, JAPAN

## Abstract

**Introduction:**

Carotid artery stenosis is one of the leading causes of ischemic stroke. Carotid artery stenting has become well-established as an effective treatment option for carotid artery stenosis. For this study, we aimed to determine the efficacy and safety of carotid stenting in a population-based large cohort of patients by analyzing the Taiwan National Healthcare Insurance (NHI) database.

**Methods:**

2,849 patients who received carotid artery stents in the NHI database from 2004 to 2010 were identified. We analyzed the risk factors of outcomes including major adverse cardiovascular events including death, acute myocardial infarction, and cerebral vascular accidents at 30 days, 1 year, and overall period and further evaluated cause of death after carotid artery stenting.

**Results:**

The periprocedural stroke rate was 2.7% and the recurrent stroke rate for the overall follow-up period was 20.3%. Male, diabetes mellitus, and heart failure were significant risk factors for overall recurrent stroke (Hazard Ratio (*HR*) = 1.35, *p* = 0.006; *HR* = 1.23, *p* = 0.014; *HR* = 1.61, *p* < 0.001, respectively). The periprocedural acute myocardial infarction rate was 0.3%. Age and Diabetes mellitus were the significant factors to predict periprocedural myocardial infarction (*HR = 3.06*, *p = 0.019; HR* = 1.68, *p < *0.001, respectively). Periprocedural and overall mortality rates were 1.9% and 17.3%, respectively. The most significant periprocedural mortality risk factor was acute renal failure. Age, diabetes mellitus, acute or chronic renal failure, heart failure, liver disease, and malignancy were factors correlated to the overall period mortality.

**Conclusion:**

Periprocedural acute renal failure significantly increased the mortality rate and the number of major adverse cardiovascular events, and the predict power persisted more than one year after the procedure. Age and diabetes mellitus were significant risk factors to predict acute myocardial infarction after carotid artery stenting.

## Introduction

Carotid artery stenosis is a correctable etiology of ischemic stroke, which is one of the leading causes of death and permanent disability [1–3]. The degree of stenosis, previous silent myocardial infarctions, contralateral obstruction, local arterial collateralization, and cardiovascular risk factors each play a significant role in the development of a stroke. The prevalence of carotid stenosis has been reported as approximately 0.5% for people over the age of 60 and 10% for those over 80, and 5% to 12% of all stroke patients are estimated to have significant internal carotid artery stenosis [4–6].

Carotid artery endarterectomy and stenting has been established as an effective treatment for carotid stenosis [7, 8]. Though carotid artery endarterectomy successfully reduces the risk of recurrent stroke in patients with severe carotid stenosis, carotid artery stenting is an optional treatment as an alternative to carotid artery endarterectomy, especially for patients with more comorbidities and a higher surgical risk.

In the *Carotid and Vertebral Transluminal Angioplasty Study* (CAVATAS) conducted in 2001 the carotid artery stenting group was shown to have similar stroke and mortality outcomes to those of the endarterectomy group [9]. Later in 2004, the long-awaited results of the *Stenting and Angioplasty with Protection in Patients at High Risk for Endarterectomy* (SAPPHIRE) study revealed the non-inferior outcomes of carotid artery stenting when compared to carotid endarterectomy [10]. In addition, significantly lower periprocedural myocardial infarction rates in other studies point to the positive qualities of carotid artery stenting [11, 12]. The *Carotid Revascularization Endarterectomy vs*. *Stenting Trial* (CREST), providing data on 2,502 patients with a median follow-up period of 2.5 years, found no difference in the primary outcomes between the stenting and endarterectomy groups [13]. However, a higher risk of stroke and a lower risk of myocardial infarction were noted in the stenting group during the periprocedural period.

Although carotid artery stenting has been one of the mainstream treatments for symptomatic carotid stenosis; however, a large-scale population survey for post-procedure long-term major adverse cardiovascular events has, to date, not been completed. We analyzed the Taiwan National Health Insurance Research Database (NHIRD) for the outcome of carotid artery stenting. The NHIRD was implemented in 1995 and contains the healthcare information of over 99% of Taiwanese citizens. All contracted medical institutions must submit standard computerized claim documents for medical expenses. By analyzing the NHIRD, we designed a national retrospective study to analyze the efficacy, safety, and the risk factors of major adverse cardiovascular events after carotid artery stenting for an Asian population.

## Methods

### Database

The National Health Insurance (NHI) program has provided compulsory universal health insurance in Taiwan since 1995 and covers more than 99% of Taiwan’s population of more than 22 million people (http://nhird.nhri.org.tw/en/). Patient identification numbers, gender, birthday, dates of admission and discharge, medical institutions providing the services, the ICD-9-CM (International Classification of Diseases, 9th Revision, Clinical Modification) diagnostic and procedure codes (up to five each), and outcomes are encrypted. Researchers wanting to use the NHIRD and its data subsets need to sign a written agreement declaring that they have no intention of attempting to obtain information that could potentially violate the privacy of patients or care providers. We conducted a nationwide population-based cohort study using the NHIRD, which consists of standard computerized claims documents submitted by medical institutions under contract with the NHI Program for reimbursement of medical expenses. The accuracy of diagnoses in the NHIRD for major diseases such as stroke and acute coronary syndrome has previously been validated [14–16].

This study was approved by the Ethics Institutional Review Board of Chang Gung Memorial Hospital.

### Patient identification

We enrolled patients in the NHIRD who received carotid artery stenting from 2004 to 2010 and defined index hospitalization as first time admission for carotid artery stenting. According to the NHI Guide, carotid artery stenting is to be carried out for symptomatic patients with carotid stenosis ≥ 60% or asymptomatic patients with carotid stenosis ≥ 80%. The exclusion criteria are patients with head and neck cancer and/or patients under the age of 18. Patients were followed until death or the end of the study period (2010). Death was defined as withdrawal of the patient from the NHI program. Causes of death were defined according to the primary diagnosis of hospitalization in the three months preceding death [17]. Primary outcomes were major adverse cardiovascular events (MACE), including death, acute myocardial infarction, and cerebral vascular accident. The median number of annual carotid artery stenting cases of each hospital was 43, and we defined a high volume center as a hospital that performed 43 or more cases of carotid stent annually. The periprocedural period was defined as the 30-day period after the procedure.

Comorbidities, listed by ICD-9 codes, included acute myocardial infarction (410), ischemic cerebrovascular accident (430–432), hemorrhagic cerebrovascular accident (433–434), transient ischemic attack (435), unspecified stroke (436), diabetes mellitus (250), acute renal failure (584), chronic kidney disease (585–586), hypertension (401–405), dyslipidemia (272), gout (274), atrial fibrillation (427.31), liver disease (456.0–456.21, 571.2, 571.4–571.6, 572.2–572.8), chronic obstructive pulmonary disease (490–492, 494, 496), and heart failure (428). In addition, Charlson comorbidity index (CCI) was calculated according to a previous study [18].

### Statistical analysis

The clinical characteristics of patients are expressed in numerical form, categorical variables as a percentages and continuous variables using mean and standard deviations. The primary goal of this study was to determine whether a patient's clinical characteristics are associated with predefined adverse outcomes (i.e., stroke, AMI, all-cause mortality, and MACE). The association between those time-to-event-outcomes (prognoses) and clinical characteristics was investigated using multivariate Cox regression analysis with forward stepwise selection. The results are presented as adjusted hazard ratios (*HR*) with corresponding 95% confidence intervals (CIs). All data analysis was conducted using SPSS software version 15 (SPSS Inc., Chicago, IL, USA).

## Results

### Demographics and clinical characteristics

A total of 3,106 patients who received carotid artery stenting (CAS) between 2004 and 2010 were identified. Before index admission, 229 patients diagnosed with cancers of the head and neck (ICD9: 140–150) and 28 patients under the age of 18 at index admission were excluded, resulting in a total of 2,849 adult patients. The average duration of follow-up was 903 days (2.47 years) with a standard deviation of 664 days (1.82 years).

In the whole cohort, the mean age was 70.3 years (*SD* = 10.8) and 2,230 (78.3%) of the patients were male. Of the patients receiving CAS, 41.8% had diabetes mellitus, 92.2% had hypertension requiring medication control, and 55.4% had dyslipidemia. Other clinical characteristics and comorbid conditions of the entire cohort population are listed in Table 1. In terms of previous strokes, 2,698 patients (94.7%) had had a stroke diagnosed prior to the CAS, the majority (92.5%) of which comprised ischemic strokes, and only 4.6% of patients had a history of hemorrhagic strokes prior to the procedure. The mean score of the Charlson comorbidity index was 2.89 (*SD* = 2.08) with a range of 0 to 15 (Table 1).

**Table 1:** Baseline clinical characteristics at index carotid artery stenting (*N* = 2,849).

Variable	Number (%) or M ± SD
Age, year (range)	70.3 ± 10.8 (19.5–96.2)
<60 years old	469 (16.5)
60–69 years old	717 (25.2)
70–79 years old	1,172 (41.1)
≥80 years old	491 (17.2)
Gender, male	2,230 (78.3)
Underlying disease	
Diabetes mellitus	1,190 (41.8)
Hypertension	2,628 (92.2)
Acute renal failure	86 (3.0)
Chronic kidney disease	164 (5.8)
Gout	272 (9.5)
Dyslipidemia	1,578 (55.4)
Atrial fibrillation	162 (5.7)
Heart failure	275 (9.7)
Malignancy^†^	202 (7.1)
Chronic obstructive pulmonary disease	343 (12.0)
Chronic liver disease	183 (6.4)
Charlson comorbidity index (CCI) (range)	2.89 ± 2.08 (0–15)
Myocardial infarction	281 (9.9)
Previous stroke	
Any stroke‡	2,698 (94.7)
Ischemic stroke	2,635 (92.5)
Transient ischemic attack	572 (20.1)
Hemorrhagic stroke	131 (4.6)
Other stroke (unspecified)	119 (4.2)

† Patients with head and neck cancer were not included in this study;

‡ A discrepancy may exist between the sum of the subgroups and the total as a result of a single patient having had two or more strokes.

### Recurrent strokes

Table 2 shows the association between clinical characteristics and post stenting strokes. The recurrent stroke rate was 2.7% (77 events) during the periprocedural period, 13.1% (374 events) at 1 year, and 20.3% (578 events) for the overall follow-up period. The results show that age was not associated with post stenting stroke at any of the follow-up periods. Male patients had a higher risk of stroke at 1 year and overall (*HR* = 1.33, *p* = 0.035; *HR* = 1.35 *p* = 0.006). Patients with heart failure also showed a trend toward higher risk at overall follow-up (*HR* = 1.61 *p* < 0.001). Though the risk of recurrent stroke was not significantly higher for patients with diabetes mellitus during short-term follow-up, the risk was significantly higher for the long-term follow-up period (*HR* = 1.23, *p* = 0.014 for the overall period). As can be seen in Table 2, chronic kidney disease was associated with lower risks of long-term stroke (*HR* = 0.51, *p* = 0.004). In addition, receiving CAS in a high volume hospital or center provided a protective effect against stroke risk at 1 year (*HR* = 0.74, *p* = 0.005) and for the overall period (*HR* = 0.84, *p* = 0.036).

**Table 2:** Factors associated with any stroke after index carotid artery stenting (multivariate Cox regression with forward stepwise selection).

	Outcome: any stroke					
	30 days		1 year		Overall	
	(77 events, 2.7%)		(374 events 13.1%)		(578 events, 20.3%)	
Variable	HR (95% CI)	*P*	HR (95% CI)	*P*	HR (95% CI)	*P*
Age (per decade)	–	–	–	–	–	–
Gender, male	–	–	1.33 (1.02–1.74)	0.035	1.35 (1.09–1.68)	0.006
Diabetes	–	–	–	–	1.23 (1.04–1.45)	0.014
Hypertension	–	–	–	–	–	–
ARF	–	–	–	–	–	–
Chronic kidney disease	–	–	–	–	0.51 (0.32–0.81)	0.004
Gout	–	–	–	–	–	–
Dyslipidemia	–	–	–	–	–	–
Atrial fibrillation	–	–	1.53 (1.06–2.21)	0.023	–	–
Heart failure	–	–	–	–	1.61 (1.25–2.07)	<0.001
Malignancy	–	–	–	–	–	–
COPD	–	–	–	–	–	–
Liver disease	–	–	–	–	–	–
High volume center†	–	–	0.74 (0.60–0.91)	0.005	0.84 (0.71–0.99)	0.036
Medical center	–	–	–	–	–	–

ARF = acute renal failure; COPD = chronic obstructive pulmonary disease; HR = hazard ratio; CI = confidence interval;

† defined as ≥ 43 volume per year;

The model was fully adjusted for the listed variables.

### Acute myocardial infarction

We analyzed the incidence of acute myocardial infarctions (AMI) after CAS in order to identify related risk factors. A total of 0.3% of patients (8 events) developed AMI during the periprocedural period, 1.5% of patients (43 events) at one year, and 4.0% of patients (113 events) for the overall period. Diabetes played a significant role in AMI occurring after CAS during the periprocedural period (*HR* = 4.87, *p* = 0.053), at one year (*HR* = 2.23, *p* = 0.010), and for overall (*HR* = 1.78, *p* = 0.003). Aside from diabetes, age also indicated a higher risk for AMI within 30 days after CAS (*HR* = 3.06, *p* = 0.019), 1 year (*HR* = 1.74, *p* = 0.003) and throughout the overall period (*HR* = 1.67, *p* < 0.001). Furthermore, heart failure was also associated with a higher risk of AMI after CAS for the overall period (*HR* = 2.34, *p* < 0.001) (see Table 3).

**Table 3:** Factors associated with acute myocardial infarction after index carotid artery stenting (multivariate Cox regression with forward stepwise selection).

	Outcome: acute myocardial infarction						
	30 days		1 year		Overall		
	(8 events, 0.3%)		(43 events, 1.5%)		(113 events, 4.0%)		
Variables	HR (95% CI)	*P*	HR (95% CI)	*P*	HR (95% CI)		*P*
Age (per decade)	3.06 (1.20–7.76)	0.019	1.74 (1.21–2.50)	0.003	1.67 (1.34–2.10)		<0.001
Gender, male	–	–	–	–	–		–
Diabetes	4.87 (0.98–24.21)	0.053	2.23 (1.21–4.10)	0.010	1.78 (1.22–2.60)		0.003
Hypertension	–	–	–	–	–		–
ARF	–	–	–	–	–		–
Chronic kidney disease	–	–	–	–	–		–
Gout	–	–	2.44 (1.17–5.10)	0.017	–		–
Dyslipidemia	–	–	–	–	–		–
Atrial fibrillation	–	–	–	–	–		–
Heart failure	–	–	–	–	2.34 (1.48–3.71)		<0.001
Malignancy	–	–	–	–	–		–
COPD	–	–	–	–	–		–
Liver disease	–	–	–	–	–		–
High volume center†	–	–	–	–	–		–
Medical center	–	–	–	–	–	–	

ARF = acute renal failure; COPD = chronic obstructive pulmonary disease; HR = hazard ratio; CI = confidence interval; NA = not applicable;

† defined as ≥43 volume per year;

The model was fully adjusted for the listed variables.

### All-cause mortality

Table 4 shows the relationship between clinical characteristics and all-cause mortality following CAS. The mortality rate was 1.9% (54 events) during the periprocedural period, 7.4% (210 event) at 1 year, and 17.3% (492 events) for the overall follow-up period. Acute renal failure during the index admission was a significant predictor of all-cause mortality perioperatively, at 1 year, and for the overall period (*HR* = 5.79, *p* < 0.001; *HR* = 3.56, *p* < 0.001; *HR* = 1.75, *p* = 0.005, respectively). The presence of diabetes, heart failure, malignancy, and liver disease were also associated with a higher mortality rate at 1 year (*HR* = 1.37, *p* = 0.028; *HR* = 1.74, *p* = 0.003; *HR* = 1.93, *p* = 0.001; *HR* = 1.55, *p* = 0.049, respectively) and during the overall period (*HR* = 1.64, *p* < 0.001; *HR* = 1.63, *p* < 0.001; *HR* = 2.27, *p* < 0.001; *HR* = 1.63, *p* = 0.001, respectively). In addition, advanced age and the presence of chronic kidney disease were likewise associated with a higher risk of mortality across the overall period (*HR* = 1.37, *p* < 0.001; *HR* = 1.80, *p* < 0.001). Interestingly, dyslipidemia was found to be a protective risk factor. We will discuss this anomaly below.

**Table 4:** Factors associated with all cause mortality after the index carotid artery stenting (multivariate Cox regression with forward stepwise selection).

	Outcome: all cause mortality					
	30 days		1 year		Overall	
	(54 events, 1.9%)		(210 events, 7.4%)		(492 events, 17.3%)	
Variable	HR (95% CI)	*P*	HR (95% CI)	*P*	HR (95% CI)	*P*
Age (per decade)	0.80 (0.65–0.99)	0.038	–	–	1.37 (1.24–1.52)	<0.001
Gender, male	–	–	–	–	–	–
Diabetes	–	–	1.37 (1.04–1.82)	0.028	1.64 (1.37–1.97)	<0.001
Hypertension	–	–	–	–	–	–
ARF	5.79 (2.60–12.86)	<0.001	3.56 (2.26–5.60)	<0.001	1.75 (1.19–2.58)	0.005
Chronic kidney disease	–	–	–	–	1.80 (1.33–2.43)	<0.001
Gout	0.17 (0.02–1.22)	0.078	–	–	–	–
Dyslipidemia	0.57 (0.33–0.99)	0.046	0.63 (0.48–0.83)	0.001	0.77 (0.64–0.92)	0.004
Atrial fibrillation	–	–	–	–	–	–
Heart failure	–	–	1.74 (1.20–2.51)	0.003	1.63 (1.27–2.09)	<0.001
Malignancy	–	–	1.93 (1.29–2.88)	0.001	2.27 (1.75–2.95)	<0.001
COPD	–	–	–	–	–	–
Liver disease	–	–	1.55 (1.01–2.39)	0.049	1.63 (1.22–2.18)	0.001
High volume center†	–	–	–	–	–	–
Medical center	–	–	–	–	–	–

ARF = acute renal failure; COPD = chronic obstructive pulmonary disease; HR = hazard ratio; CI = confidence interval;

† defined as ≥ 43 volume per year;

The model was fully adjusted for the listed variables.

### Major adverse cardiovascular events

We defined Major Adverse Cardiovascular Events (MACE) as stroke, acute myocardial infarction, or death. Our results (Table 5) suggest that acute renal failure is a strong predictor of mortality at 30 days, 1 year, and for the overall period (*HR* = 3.60, *p* = 0.001; *HR* = 1.91, *p* < 0.001; *HR* = 1.49, *p* = 0.010, respectively). Similarly, diabetes, heart failure, and malignancy were associated MACE at 1 year (*HR* = 1.21, *p* = 0.027; *HR* = 1.40, *p* = 0.007; *HR* = 1.37, *p* = 0.026, respectively) and during the overall period (*HR* = 1.36, *p* < 0.001; *HR* = 1.66, *p* < 0.001; *HR* = 1.31, *p* = 0.019, respectively). In addition, older age, male sex, and liver disease were associated with MACE during the overall period (*HR* = 1.19, *p* < 0.001; *HR* = 1.25, *p* = 0.005; *HR* = 1.31, *p* = 0.020). Receiving CAS in a high volume hospital or medical center provided a protective effect against MACE risk at 1 year (*HR* = 0.80, *p* = 0.007).

**Table 5:** Factors associated with major adverse cardiovascular events after index carotid artery stenting (multivariate Cox regression with forward stepwise selection).

	Outcome: major adverse cardiovascular event					
	30 days		1 year		Overall	
	(134 events, 4.7%)		(580 events, 20.4%)		(988 events, 34.7%)	
Variable	HR (95% CI)	*P*	HR (95% CI)	*P*	HR (95% CI)	*P*
Age (per decade)	–	–	1.09 (1.00–1.18)	0.045	1.19 (1.12–1.27)	<0.001
Gender, male	–	–	–	–	1.25 (1.07–1.47)	0.005
Diabetes	–	–	1.21 (1.02–1.43)	0.027	1.36 (1.20–1.55)	<0.001
Hypertension	–	–	–	–	–	–
ARF	3.60 (1.75–7.42)	0.001	1.91 (1.33–2.74)	<0.001	1.49 (1.10–2.03)	0.010
Chronic kidney disease	0.30 (0.10–0.88)	0.028	–	–	–	–
Gout	–	–	–	–	–	–
Dyslipidemia	–	–	–	–	–	–
Atrial fibrillation	–	–	–	–	–	–
Heart failure	–	–	1.40 (1.09–1.78)	0.007	1.66 (1.39–1.99)	<0.001
Malignancy	–	–	1.37 (1.04–1.81)	0.026	1.31 (1.04–1.63)	0.019
COPD	–	–	–	–	–	–
Liver disease	–	–	–	–	1.31 (1.04–1.65)	0.020
High volume center†	–	–	0.80 (0.68–0.94)	0.007	–	–
Medical center	–	–	–	–	–	–

ARF = acute renal failure; COPD = chronic obstructive pulmonary disease; HR = hazard ratio; CI = confidence interval;

† defined as ≥ 43 volume per year;

The model was fully adjusted for the listed variables.

### Causes of mortality

A total of 492 patients in the database were identified as deceased, 386 of which were hospitalized within 90 days prior to the reported date of death. The causes of mortality are listed in Table 6. The primary diagnosis during the last admission period appeared to be related to the patients’ death, with the leading causes of death being sepsis (23.3%), followed by cardiovascular disease (18.1%), ischemic cerebral vascular disease (16.3%), malignancy (14.5%), and respiratory failure (9.3%).

**Table 6:** Causes of mortality (*n* = 386).

Cause of mortality	Number	Percentage
Sepsis	91	23.3
Cardiovascular disease	69	18.1
Ischemic cerebral vascular disease	63	16.3
Malignancy	56	14.5
Respiratory failure	36	9.3
Cannot be specified (other)	31	8.0
Hemorrhagic cerebral vascular disease	16	4.1
Gastrointestinal bleeding	8	2.1
Peripheral artery disease	7	1.8
Renal failure	6	1.6
Diabetes mellitus	3	0.8

### Additional analysis

The multivariate analyses were stratified by gender and the results for male and female patients are shown in Supplemental Table A and Supplemental Table B in the supporting file S1 Appendix, respectively. In addition to the stepwise “reduced models”, the fully adjusted “enter method” maximal models for identifying associated factors with various outcomes are presented at Supplemental Tables C to F in the S1 Appendix file.

## Discussion

Our study demonstrates real-world short- and long-term outcomes of CAS in an East Asian population and provides a comparison of these results with previous clinical studies. In our study, the periprocedural MACE rate was 4.7%, which is comparable to previous findings in a global survey of CAS (6.29%) [19] and in the CREST study (5.2%). We observed periprocedural stroke, myocardial infarction, and mortality rates of 2.7%, 0.3%, and 1.9%e, which compare to those from the CREST study of 4.1%, 1.1%, and 0.7%, respectively. However, the long-term follow-up result from our study showed that approximately one-fifth of patients suffered from recurrent stroke or mortality in the median 2.2 year follow-up period.

### Recurrent Stroke

Perioperative stroke is the first major issue to be stressed for carotid artery intervention. The global survey published in 2000 reported that during the 30-day periprocedural period for carotid artery intervention, 2.57% of the cases had TIA, 2.48% minor stroke, and 1.36% major stroke [19]. Other studies suggest that CAS may share a higher risk than CEA for carotid artery disease [13, 20]. In our study, the periprocedural stroke rate was 2.7%,which is between the event rate of CAS and CEA in the CREST study (4.1% and 2.3%, respectively). In terms of long-term follow-up, the recurrent stroke rate rose to 20.3%, and we found significantly higher recurrent stroke rates among male, diabetic, and heart failure patients. Our findings were double the long-term results of the CREST study, which recorded a 10.2% recurrent stroke rate in a median 2.5 year follow-up duration. However, the CREST study [21] excluded patients with atrial fibrillation, dialysis dependent chronic kidney disease, malignancy, and/or other underlying conditions making for surgery unsuitable, whereas our real-world study included such patients. Hence, our cohort data may better reflect conditions in the real clinical world. Moreover, the greater number of patients in our study compared to the CREST study who were of an advanced aged (70.3 years vs. 68.9 years), presented with diabetes mellitus (41.8% vs. 30.6%), and presented with hypertension (92.2% vs. 85.8%) could have also affected the long-term results.

In analyzing the risk of recurrent stroke, we found that patients with a diagnosis of chronic kidney disease had a protective effect against stroke, and this trend reached significance for long-term statistical results (*HR* = 0.51, *p* = 0.004). However, chronic kidney disease is a well-established risk factor for stroke [22, 23], and we attribute this paradoxical result to the effect of competitive outcomes of higher mortality in the chronic kidney disease group. Chronic kidney disease was also found to be a significant risk factor of mortality in our long-term follow-up.

### Acute Myocardial infarction

Coronary artery disease is known to be correlated with carotid artery disease [24, 25], and periprocedural myocardial infarction is an important concern related to the performance of carotid artery intervention, regardless of whether endarterectomy or the stenting method is adopted. Periprocedural myocardial infarction has long been discussed in comparison to the adverse outcome between CAS and CEA, and CAS is thought to reduce the rate of periprocedural myocardial infarction. The results of our study showed a 0.3% incidence of acute myocardial infarction during the periprocedural period, which is much lower than the 1.0% found in the symptomatic group after carotid stenting in the CREST study. We hypothesize that the rate of periprocedural myocardial infarction might be lower among East Asian populations. Studies have revealed a lower incidence of coronary artery disease among Japanese heart failure patients compared to patients in Western countries [26]. Moreover, different rates of myocardial infarction between different ethnicities have been evidenced in Singapore [27]. The rate of lower coronary heart disease in East Asian populations has been discussed for quite some time now, and at present this phenomenon is primarily attributed to a lower level of serum total cholesterol and a lower smoking rate [28]. Though conventional risk factors may constitute a partial explanation, another study also showed that rates of myocardial infarction differ between white, South Asian, and Afro-Caribbean patients after adjusting for risk factors and social class [29]. Further investigation is warranted to investigate whether other genetic causes result in the lower periprocedural myocardial infarction rate among people of East Asian descent.

In the risk analysis for myocardial infarction, age and diabetes mellitus—which is a known coronary heart disease equivalent—constituted significant risk factors for acute myocardial infarction occurring in our periprocedural period (*HR* = 3.06, *p* = 0.019; *HR* = 4.87, *p* = 0.053, respectively). In our long-term follow-up, 4.0% of participants suffered myocardial infarction during the study period. Age (*HR* = 1.67, *p <* 0.001), diabetes (*HR* = 1.78, *p* = 0.003), and heart failure (*HR* = 2.34, *p < 0*.001) all contributed to a significantly increased risk of acute myocardial infarction.

### Mortality

The periprocedural mortality rate was 1.9% in our finding compared to 0.7% in the CREST study. The mortality rate was significantly elevated for patients who experienced acute renal failure during the periprocedural period (*HR* 5.79, *p* < 0.001). This result also held true across longer time periods. Periprocedural acute renal failure has been known to be a strong indicator for death in previous observations and studies [30, 31], and our result also support this conclusion. Critical patients may experience many different scenarios that cause acute renal failure, including sepsis, dehydration, shock, or medication related nephropathy. Contrast-induced nephropathy is one of the possible causes of acute renal failure for the percutaneous intervention, and studies have described its possible etiology and prevention strategies [32, 33]; a proper strategy should be planned to prevent this from happening. Furthermore, in the overall follow-up period, age, DM, CKD, heart failure, and underlying malignancy were all significant risk factors for mortality, which is consistent with the previous studies [34–36]. For the overall follow-up period, our database showed higher death rates compared to the CREST study (17.3% vs. 11.3%, respectively). However, as discussed above, in contrast to the CREST study, we only excluded younger patients and patients with head and neck cancer; we also incorporated several different patient characteristics. Taken together, these differences with the CREST study likely explain the long-term outcomes of our study.

We further analyzed the cause of mortality of the overall period. Infection-related causes comprised the majority in the analysis, and most patients in this group were diagnosed as pneumonia- or respiratory-system-related mortality. Cardiovascular diseases, including myocardial infarction and heart failure, occupied the second position. Following cerebral infarction, respiratory and cardiovascular disease were the leading causes of mortality at the long-term follow-up [37]. In order to decrease long-term mortality, it is important to carefully evaluate the patient’s underlying condition including respiratory system, cough function, coronary artery disease, and heart failure. Cautious selection of the patients for CAS and appropriate attention to the evaluation and management of the patient’s underlying condition should be ensured in order to decrease the overall mortality.

## Limitations

This study had several limitations. First, the diagnoses of comorbidities we collected were from claims data and ICD-9-CM codes, and we were unable to distinguish underlying disease severity from the current coding system. Because we could not differentiate between minor and major CVA events—and the severity of recurrent stroke could be an important factor in peri- and post-procedure complications and outcome evaluation—it is possible that this resulted in a relatively high long-term recurrent stroke rate in our database. In addition, coding errors may exist in the database used for this study, and there was no way we could verify the accuracy of the data collected from the NHIRD. Second, we were unable to take into account personal information of patients such as lifestyle, family history, body mass index, smoking status, and laboratory data, all of which can affect survival and stroke rates. Third, we did not enroll patients who did not receive CAS as a control group to compare the results of stenting, medical treatment alone, or surgical endarterectomy; hence, we were unable to demonstrate a comprehensive picture of people in modern society with symptomatic carotid stenosis without CAD for this study. Finally, the risk factor of dyslipidemia was found to be protective against mortality in our study. The limitation of the space for the coding in the NHI database could be one possible explanation for this anomaly. For example, a patient with a critical illness could have more severe comorbidities, and since diagnosis space is limited, the ranking of a dyslipidemia diagnosis could effectively push it off the list of diagnoses, resulting in the apparent “protective effect” of dyslipidemia against mortality.

## Conclusion

In Taiwan, carotid artery stenting for carotid stenosis shares a similar periprocedural outcome with other current large-scale trials. In terms of current percutaneous intervention techniques, carotid artery stenting remains a relative risky procedure. In addition, we found that it was common for patients who suffer from carotid artery disease to have more comorbidities, which could lead to post-procedure complications or adverse events. Age and diabetes mellitus were significant risk factors to predict acute myocardial infarction after carotid artery stenting. Acute renal failure during the periprocedural period significantly increased the mortality rate and the number of major adverse cardiovascular events, and the predict power persisted more than one year after the procedure. Therefore, caution should be exercised when selecting patients to undergo carotid artery stenting and appropriate steps should be taken to prevent adverse effects.

## Supporting Information

S1 Appendix Supplemental table A: The associated factors with primary outcomes in men subjects. Supplemental table B: The associated factors with primary outcomes in women subjects. Supplemental table C: Full model of factors associated with any stroke. Supplemental table D: Full model of factors associated with acute myocardial infarction. Supplemental table E: Full model of factors associated with all cause mortality. Supplemental table F: Full model of factors associated with major adverse cardiovascular events.
(DOCX)
