# Supplementary material for: Short- and Long-Term Major Cardiovascular Adverse Events in Carotid Artery Interventions: A Nationwide Population-Based Cohort Study in Taiwan
Source: PLoS One. 2015 Mar 24;10(3):e0121016. doi: 10.1371/journal.pone.0121016 (PMC4372443; doi:10.1371/journal.pone.0121016)
Supplement: S1 Appendix — Supplemental table B: The associated factors with primary outcomes in women subjects. Supplemental table C: Full model of factors associated with any stroke. Supplemental table D: Full model of factors associated with acute myocardial infarction. Supplemental table E: Full model of factors associated with all cause mortality. Supplemental table F: Full model of factors associated with major adverse cardiovascular events. (DOCX) [file pone.0121016.s001.docx]

Supplemental table 1: The associated factors with primary outcomes in men subjects

| Outcome / period / event (%) | HR (95% CI) | P |
| --- | --- | --- |
| Stroke at 30 days (67 events, 3.0%) | - (none) | - (none) |
| Stroke at all-course (476 events, 21.3%) |  |  |
| Diabetes | 1.29 (1.07–1.54) | 0.007 |
| Chronic kidney disease | 0.42 (0.24–0.73) | 0.002 |
| Heart failure | 1.49 (1.11–1.99) | 0.007 |
| High volume center† | 0.83 (0.69–1.00) | 0.048 |
| AMI at 30 days (7 events, 0.3%) | - (none) | - (none) |
| AMI at all-course (90 events, 4.0%) |  |  |
| Age (per decade) | 1.79 (1.38–2.31) | <0.001 |
| Diabetes | 1.62 (1.06–2.48) | 0.024 |
| Heart failure | 2.41 (1.43–4.07) | 0.001 |
| All-cause mortality at 30 days (43 events, 1.9%) |  |  |
| Age (per decade) | 0.76 (0.59–0.97) | 0.028 |
| ARF | 5.26 (2.21–12.51) | <0.001 |
| All-cause mortality at all-course (397 events, 17.8%) |  |  |
| Age (per decade) | 1.36 (1.21–1.52) | <0.001 |
| Diabetes | 1.64 (1.34–2.01) | <0.001 |
| ARF | 1.85 (1.21–2.85) | 0.005 |
| Chronic kidney disease | 1.68 (1.20–2.35) | 0.003 |
| Dyslipidemia | 0.81 (0.66–0.99) | 0.037 |
| Heart failure | 1.54 (1.15–2.06) | 0.004 |
| Malignancy | 2.59 (1.96–3.44) | <0.001 |
| Liver disease | 1.53 (1.09–2.14) | 0.013 |
| MACE at 30 days (112 events, 5.0%) |  |  |
| ARF | 2.35 (1.14–4.82) | 0.020 |
| MACE at all-course (799 events, 35.8%) |  |  |
| Age (per decade) | 1.20 (1.11–1.29) | <0.001 |
| Diabetes | 1.38 (1.20–1.59) | <0.001 |
| ARF | 1.43 (1.02–2.00) | 0.040 |
| Heart failure | 1.58 (1.28–1.94) | <0.001 |
| Malignancy | 1.43 (1.13–1.82) | 0.003 |

ARF = acute renal failure; COPD = chronic obstructive pulmonary disease; HR = hazard ratio; CI = confidence interval; AMI = acute myocardial infarction; MACE = major adverse cardiovascular events;

† defined as ≥ 43 volume per year;

The model was fully adjusted for the listed variables.

Supplemental table 2: The associated factors with primary outcomes in women subjects

| Outcome / period / event (%) | HR (95% CI) | P |
| --- | --- | --- |
| Stroke at 30 days (10 events, 1.6%) | - (none) | - (none) |
| Stroke at all-course (102 events, 16.5%) |  |  |
| Heart failure | 2.04 (1.24–3.35) | 0.005 |
| Liver disease | 1.86 (1.02–3.40) | 0.043 |
| AMI at 30 days (1 event, 0.2%) | - (none) | - (none) |
| AMI at all-course (23 events, 3.7%) |  |  |
| Diabetes | 3.11 (1.20–8.03) | 0.019 |
| Heart failure | 2.58 (1.00–6.68) | 0.050 |
| All-cause mortality at 30 days (11 events, 1.8%) | - (none) | - (none) |
| All-cause mortality at all-course (95 events, 15.3%) |  |  |
| Age (per decade) | 1.33 (1.06–1.68) | 0.015 |
| Diabetes | 1.67 (1.08–2.59) | 0.022 |
| Chronic kidney disease | 2.34 (1.28–4.30) | 0.006 |
| Heart failure | 2.00 (1.18–3.39) | 0.010 |
| Liver disease | 2.14 (1.19–3.86) | 0.011 |
| MACE at 30 days (22 events, 3.6%) | - (none) | - (none) |
| MACE at all-course (189 events, 30.5%) |  |  |
| Hypertension | 2.85 (1.17–6.95) | 0.021 |
| Heart failure | 2.43 (1.71–3.44) | <0.001 |
| Liver disease | 1.95 (1.26–3.02) | 0.003 |

HR = hazard ratio; CI = confidence interval; AMI = acute myocardial infarction; MACE = major adverse cardiovascular events;

The model was fully adjusted for the listed variables.

Supplemental table 3: Full model of factors associated with any stroke

|  | Outcome: any stroke | | | | | | | |
| --- | --- | --- | --- | --- | --- | --- | --- | --- |
|  | 30 days | |  | 1 year | |  | Overall | |
|  | (77 events, 2.7%) | |  | (374 events 13.1%) | |  | (578 events, 20.3%) | |
| Variable | HR (95% CI) | *P* |  | HR (95% CI) | *P* |  | HR (95% CI) | *P* |
| Age (per decade) | 0.98 (0.79–1.22) | 0.847 |  | 1.06 (0.96–1.18) | 0.239 |  | 1.08 (0.99–1.17) | 0.081 |
| Gender, male | 1.88 (0.96–3.66) | 0.066 |  | 1.36 (1.04–1.79) | 0.025 |  | 1.36 (1.09–1.69) | 0.005 |
| Diabetes | 0.98 (0.62–1.57) | 0.939 |  | 1.11 (0.90–1.37) | 0.321 |  | 1.22 (1.03–1.45) | 0.021 |
| Hypertension | 0.81 (0.36–1.79) | 0.600 |  | 1.00 (0.67–1.50) | 0.987 |  | 1.03 (0.75–1.42) | 0.859 |
| ARF | 1.00 (0.23–4.45) | 0.996 |  | 0.84 (0.40–1.78) | 0.653 |  | 1.14 (0.64–2.02) | 0.655 |
| Chronic kidney disease | 0.34 (0.08–1.53) | 0.161 |  | 0.60 (0.34–1.05) | 0.073 |  | 0.47 (0.29–0.77) | 0.002 |
| Gout | 1.16 (0.57–2.36) | 0.677 |  | 1.01 (0.71–1.43) | 0.956 |  | 1.00 (0.76–1.33) | 0.981 |
| Dyslipidemia | 1.33 (0.83–2.13) | 0.229 |  | 1.18 (0.95–1.45) | 0.130 |  | 1.06 (0.90–1.26) | 0.463 |
| Atrial fibrillation | 0.86 (0.30–2.41) | 0.770 |  | 1.45 (0.99–2.13) | 0.055 |  | 1.29 (0.93–1.78) | 0.127 |
| Heart failure | 1.98 (1.03–3.80) | 0.040 |  | 1.36 (0.98–1.89) | 0.067 |  | 1.47 (1.13–1.92) | 0.004 |
| Malignancy | 0.70 (0.25–1.94) | 0.494 |  | 1.01 (0.68–1.49) | 0.975 |  | 0.79 (0.55–1.14) | 0.207 |
| COPD | 1.01 (0.51–2.02) | 0.974 |  | 0.99 (0.72–1.36) | 0.956 |  | 1.06 (0.82–1.35) | 0.663 |
| Liver disease | 1.72 (0.81–3.61) | 0.155 |  | 1.24 (0.84–1.83) | 0.274 |  | 1.37 (1.01–1.87) | 0.041 |
| High volume center† | 1.04 (0.62–1.74) | 0.896 |  | 0.74 (0.58–0.95) | 0.019 |  | 0.83 (0.68–1.01) | 0.059 |
| Medical center | 1.24 (0.64–2.40) | 0.517 |  | 1.03 (0.75–1.41) | 0.868 |  | 0.97 (0.76–1.24) | 0.809 |

ARF = acute renal failure; COPD = chronic obstructive pulmonary disease; HR = hazard ratio; CI = confidence interval;

† defined as ≥ 43 volume per year.

Supplemental table 4: Full model of factors associated with acute myocardial infarction

|  | Outcome: acute myocardial infarction | | | | | | | |
| --- | --- | --- | --- | --- | --- | --- | --- | --- |
|  | 30 days | |  | 1 year | |  | Overall | |
|  | (8 events, 0.3%) | |  | (43 events, 1.5%) | |  | (113 events, 4.0%) | |
| Variables | HR (95% CI) | *P* |  | HR (95% CI) | *P* |  | HR (95% CI) | *P* |
| Age (per decade) | 2.62 (0.99–6.90) | 0.051 |  | 1.73 (1.19–2.52) | 0.004 |  | 1.67 (1.32–2.11) | <0.001 |
| Gender, male | 2.03 (0.24–17.32) | 0.519 |  | 1.56 (0.68–3.59) | 0.294 |  | 1.16 (0.73–1.86) | 0.530 |
| Diabetes | 4.96 (0.96–25.54) | 0.055 |  | 2.07 (1.10–3.88) | 0.024 |  | 1.76 (1.20–2.58) | 0.004 |
| Hypertension | NA. | NA |  | 2.36 (0.32–17.36) | 0.399 |  | 3.34 (0.82–13.63) | 0.093 |
| ARF | NA | NA |  | 0.68 (0.15–3.15) | 0.625 |  | 0.60 (0.18–2.02) | 0.413 |
| Chronic kidney disease | NA | NA |  | 1.91 (0.73–4.99) | 0.188 |  | 1.46 (0.74–2.88) | 0.274 |
| Gout | 1.59 (0.18–14.19) | 0.676 |  | 2.11 (0.98–4.51) | 0.056 |  | 1.38 (0.80–2.38) | 0.243 |
| Dyslipidemia | 0.97 (0.23–4.09) | 0.969 |  | 1.24 (0.66–2.32) | 0.506 |  | 1.09 (0.74–1.60) | 0.652 |
| Atrial fibrillation | 2.00 (0.23–17.68) | 0.531 |  | 0.74 (0.22–2.52) | 0.628 |  | 0.53 (0.23–1.24) | 0.143 |
| Heart failure | 2.16 (0.39–12.12) | 0.381 |  | 1.80 (0.79–4.06) | 0.159 |  | 2.32 (1.42–3.79) | 0.001 |
| Malignancy | 2.61 (0.49–13.90) | 0.261 |  | 0.79 (0.24–2.58) | 0.690 |  | 0.75 (0.33–1.71) | 0.493 |
| COPD | 0.57 (0.06–5.03) | 0.610 |  | 0.86 (0.37–2.02) | 0.737 |  | 1.09 (0.66–1.81) | 0.738 |
| Liver disease | 1.66 (0.18–15.16) | 0.655 |  | 1.19 (0.42–3.42) | 0.742 |  | 1.16 (0.58–2.33) | 0.669 |
| High volume center† | 0.38 (0.04–3.34) | 0.385 |  | 0.73 (0.33–1.59) | 0.422 |  | 0.88 (0.56–1.39) | 0.595 |
| Medical center | 1.05 (0.06–17.54) | 0.973 |  | 0.62 (0.26–1.49) | 0.283 |  | 0.80 (0.47–1.38) | 0.431 |

ARF = acute renal failure; COPD = chronic obstructive pulmonary disease; HR = hazard ratio; CI = confidence interval; NA = not applicable;

† defined as ≥43 volume per year.

Supplemental table 5: Full model of factors associated with all cause mortality

|  | Outcome: all cause mortality | | | | | | | |
| --- | --- | --- | --- | --- | --- | --- | --- | --- |
|  | 30 days | |  | 1 year | |  | Overall | |
|  | (54 events, 1.9%) | |  | (210 events, 7.4%) | |  | (492 events, 17.3%) | |
| Variable | HR (95% CI) | *P* |  | HR (95% CI) | *P* |  | HR (95% CI) | *P* |
| Age (per decade) | 0.78 (0.62–0.98) | 0.032 |  | 1.06 (0.92–1.21) | 0.442 |  | 1.35 (1.22–1.50) | <0.001 |
| Gender, male | 1.14 (0.58–2.23) | 0.699 |  | 0.96 (0.68–1.33) | 0.788 |  | 1.18 (0.94–1.49) | 0.151 |
| Diabetes | 1.70 (0.98–2.98) | 0.061 |  | 1.38 (1.04–1.84) | 0.027 |  | 1.65 (1.37–1.99) | <0.001 |
| Hypertension | 1.84 (0.56–6.10) | 0.316 |  | 1.03 (0.59–1.81) | 0.904 |  | 1.20 (0.80–1.78) | 0.377 |
| ARF | 8.81 (3.58–21.70) | <0.001 |  | 2.77 (1.63–4.69) | <0.001 |  | 1.77 (1.19–2.61) | 0.005 |
| Chronic kidney disease | 0.22 (0.05–1.04) | 0.056 |  | 1.56 (0.97–2.52) | 0.068 |  | 1.79 (1.32–2.43) | <0.001 |
| Gout | 0.16 (0.02–1.20) | 0.075 |  | 0.84 (0.52–1.35) | 0.471 |  | 0.84 (0.62–1.15) | 0.275 |
| Dyslipidemia | 0.50 (0.29–0.86) | 0.013 |  | 0.64 (0.49–0.85) | 0.002 |  | 0.78 (0.65–0.93) | 0.007 |
| Atrial fibrillation | 0.30 (0.04–2.26) | 0.245 |  | 1.25 (0.76–2.05) | 0.374 |  | 1.13 (0.81–1.56) | 0.481 |
| Heart failure | 1.15 (0.47–2.84) | 0.762 |  | 1.49 (1.00–2.23) | 0.052 |  | 1.61 (1.24–2.09) | <0.001 |
| Malignancy | 0.23 (0.03–1.67) | 0.146 |  | 1.87 (1.25–2.80) | 0.002 |  | 2.27 (1.75–2.95) | <0.001 |
| COPD | 1.41 (0.62–3.23) | 0.417 |  | 1.15 (0.78–1.69) | 0.493 |  | 1.00 (0.78–1.29) | 0.991 |
| Liver disease | 0.23 (0.03–1.71) | 0.153 |  | 1.60 (1.03–2.49) | 0.038 |  | 1.67 (1.24–2.24) | 0.001 |
| High volume center† | 1.36 (0.74–2.49) | 0.326 |  | 0.91 (0.66–1.25) | 0.569 |  | 0.90 (0.73–1.12) | 0.353 |
| Medical center | 1.23 (0.58–2.59) | 0.592 |  | 1.27 (0.83–1.94) | 0.264 |  | 0.99 (0.76–1.30) | 0.959 |

ARF = acute renal failure; COPD = chronic obstructive pulmonary disease; HR = hazard ratio; CI = confidence interval;

† defined as ≥ 43 volume per year.

Supplemental table 6: Full model of factors associated with major adverse cardiovascular events

|  | Outcome: major adverse cardiovascular event | | | | | | | |
| --- | --- | --- | --- | --- | --- | --- | --- | --- |
|  | 30 days | |  | 1 year | |  | Overall | |
|  | (134 events, 4.7%) | |  | (580 events, 20.4%) | |  | (988 events, 34.7%) | |
| Variable | HR (95% CI) | *P* |  | HR (95% CI) | *P* |  | HR (95% CI) | *P* |
| Age (per decade) | 0.92 (0.79–1.08) | 0.310 |  | 1.08 (0.99–1.17) | 0.085 |  | 1.17 (1.10–1.26) | <0.001 |
| Gender, male | 1.43 (0.90–2.27) | 0.126 |  | 1.22 (0.99–1.51) | 0.062 |  | 1.24 (1.06–1.46) | 0.008 |
| Diabetes | 1.30 (0.92–1.84) | 0.141 |  | 1.22 (1.03–1.45) | 0.020 |  | 1.37 (1.21–1.56) | <0.001 |
| Hypertension | 1.07 (0.55–2.08) | 0.832 |  | 1.01 (0.73–1.39) | 0.975 |  | 1.14 (0.88–1.48) | 0.335 |
| ARF | 3.16 (1.50–6.64) | 0.002 |  | 1.83 (1.23–2.73) | 0.003 |  | 1.47 (1.05–2.06) | 0.025 |
| Chronic kidney disease | 0.28 (0.09–0.82) | 0.020 |  | 1.02 (0.72–1.43) | 0.926 |  | 1.04 (0.80–1.35) | 0.777 |
| Gout | 0.72 (0.38–1.39) | 0.329 |  | 0.93 (0.71–1.24) | 0.635 |  | 0.91 (0.73–1.14) | 0.416 |
| Dyslipidemia | 0.85 (0.60–1.19) | 0.339 |  | 0.93 (0.79–1.09) | 0.368 |  | 0.92 (0.81–1.04) | 0.182 |
| Atrial fibrillation | 0.74 (0.32–1.71) | 0.481 |  | 1.32 (0.97–1.80) | 0.080 |  | 1.16 (0.91–1.48) | 0.230 |
| Heart failure | 1.55 (0.92–2.63) | 0.102 |  | 1.33 (1.03–1.73) | 0.030 |  | 1.61 (1.33–1.95) | <0.001 |
| Malignancy | 0.71 (0.33–1.52) | 0.372 |  | 1.35 (1.02–1.78) | 0.036 |  | 1.30 (1.04–1.62) | 0.023 |
| COPD | 1.17 (0.70–1.96) | 0.547 |  | 1.03 (0.81–1.32) | 0.800 |  | 1.05 (0.87–1.26) | 0.604 |
| Liver disease | 1.00 (0.51–1.99) | 0.990 |  | 1.25 (0.92–1.68) | 0.155 |  | 1.33 (1.05–1.67) | 0.016 |
| High volume center† | 1.10 (0.75–1.63) | 0.629 |  | 0.83 (0.68–1.00) | 0.056 |  | 0.89 (0.77–1.04) | 0.135 |
| Medical center | 1.22 (0.74–1.99) | 0.438 |  | 1.05 (0.82–1.35) | 0.675 |  | 1.00 (0.83–1.20) | 0.969 |

ARF = acute renal failure; COPD = chronic obstructive pulmonary disease; HR = hazard ratio; CI = confidence interval;

† defined as ≥ 43 volume per year.
